# Endoscopic screening and surveillance for gastric cancer: challenges and opportunities

**DOI:** 10.12703/r/12-17

**Published:** 2023-07-13

**Authors:** Vikneswaran Namasivayam

**Affiliations:** 1 Department of Gastroenterology and Hepatology, Singapore General Hospital; Duke NUS Medical School, Singapore

**Keywords:** Gastric intestinal metaplasia, gastric cancer screening, endoscopic screening

## Abstract

Endoscopic screening is premised on the detection of pre-symptomatic, early-stage gastric neoplasia that enables curative resection. Endoscopic screening reduces gastric cancer mortality in high-incidence countries but is highly resource-intensive. Endoscopic surveillance of high-risk subgroups of intestinal metaplasia has gained traction in low and intermediate-incidence countries, and emerging evidence suggests that risk-stratified endoscopic surveillance may facilitate timely detection of cancer. However, outcome-based evidence is required to support its adoption. Yet the impact of an endoscopy-based strategy may well lie in heralding a paradigm that regards every routine diagnostic gastroscopy as an opportunity to screen for GC. Endoscopic surveillance also renders gastric intestinal metaplasia a de facto disease, and the ramification of this needs to be further elucidated.

## Introduction

The incidence of gastric cancer (GC) has witnessed a steady decline worldwide and it is now considered a rare cancer in many parts of the world. Yet this period has also witnessed an unprecedented global interest in screening and surveillance for gastric cancer. This is driven by several trends.

GC still remains a global health challenge, with over a million new cases in 2020^1^. Despite the secular decline, an ageing population is expected to sustain the absolute number of new cases*.* The decline is uneven, with a high incidence present in several regions and within populations. Recent studies also suggest an increase in incidence among young adults below 50 years of age^2,3^.

GC is a stage-dependent disease with poor survival related to the late detection of advanced symptomatic disease in the majority of patients. The success of population-based endoscopic screening programs in East Asian countries with high GC incidence in improving outcomes by detecting early, curable cancers, as well as the increasing dissemination of endoscopic imaging and resection techniques, have motivated interest in early detection and curative endoscopic resection.

Hence, GC has joined the ranks of Barrett’s adenocarcinoma and colorectal cancer as a digestive cancer with growing advocacy for endoscopic surveillance and intervention. Yet the evidence supporting a role for endoscopic screening and surveillance in GC is still evolving, and this calls for a more nuanced approach towards the adoption of screening and surveillance strategies.

## Secondary Prevention with Endoscopic Screening and Surveillance

The long latency that precedes advanced symptomatic disease provides an opportunity for endoscopic screening and surveillance in GC. Secondary prevention consists of population-based GC screening in high-incidence countries and opportunistic surveillance of precancerous stages in target populations in countries with low-intermediate incidence.

### High-risk populations: mass endoscopic screening

There are no randomised trials on the benefits of endoscopic screening. The evidence for mass screening is largely derived from the experience of South Korea and Japan, which, despite a high GC incidence, have achieved excellent long-term survival outcomes. This has been attributed to the adoption of nationwide cancer screening programs that increase the detection of curable cancers at an early stage. Initial mass screening with radiographic modalities has been largely supplanted by endoscopic screening. 

In South Korea, gastric cancer screening with biennial upper endoscopy or upper gastrointestinal series is recommended for all Koreans of 40 years of age and above. It is heavily subsidised by the state and has a high participation rate^4^. In a nested case-control study, this intensive nationwide screening program was associated with a 21% reduction in gastric cancer mortality. The protective effect increased with the cumulative number of screening endoscopies performed, indicating a dose-response relationship between endoscopic screening and GC mortality. While endoscopy was associated with a protective effect, the upper gastrointestinal series was not protective^5^.

In Japan, radiographic screening has been practiced since the 1960s and is associated with a reduction in GC mortality^6–8^. More recently, biennial endoscopic screening has been recommended for individuals 50 years of age and above, but uptake has been constrained by manpower and resource limitations and disparities in access to endoscopy between urban and rural areas^9–11^. Besides the screening program, opportunistic endoscopic screening performed outside the screening program also contributes significantly to the detection of early GC^12^.

In a meta-analysis of observational studies (cohort and nested case-control studies) from East Asia, endoscopic screening was associated with a 40% reduction in gastric cancer mortality but not gastric cancer incidence^13^. This outcome is achieved with highly intensive screening protocols. In contrast, less frequent endoscopic screening (approximately every 4.5 years) does not appear to be effective in reducing GC mortality, as the intervals are too long for the detection of early-stage GC^14,15^. For comparison, colorectal cancer screening, which has widely accepted evidence-based screening practices, entails a yearly *stool* test or *10-yearly* colonoscopy in an average-risk individual. The logistics of regular training, accreditation, and quality assurance for screening for both endoscopists and pathologists would be considerable. Disparities in access would also need to be addressed as high-risk subgroups are often found within indigenous and migrant communities. Hence, population-based endoscopic screening does not appear to be a viable solution in low-intermediate countries. The use of non-invasive tests to improve risk stratification and the cost-effectiveness of screening remains an area of ongoing research.

### Low-Intermediate risk populations: Targeted endoscopic surveillance

There are no outcome-based studies to support endoscopic surveillance of gastric intestinal metaplasia (GIM). The practice is motivated by the goal of detecting early-stage neoplasia in high-risk patients to facilitate curative resection. Most GCs arise from a multistep cascade of gastritis, atrophic gastritis (AG), intestinal metaplasia and dysplasia. AG, GIM and dysplasia are premalignant stages with progressively increasing risks of GC^16–18^. The risk of GC is broadly comparable in magnitude to other premalignant conditions, such as Barrett’s esophagus and colon adenoma, that are recommended for endoscopic surveillance. In endoscopic surveillance, high-risk subsets of atrophic gastritis (AG) and GIM are targeted for surveillance. Dysplasia and neoplasia are targeted for curative resection.

AG refers to the loss of gastric glands, and GIM refers to the replacement of gastric epithelium by intestinal-type epithelium in response to chronic inflammation. GIM is further sub-typed into complete GIM (type I) and incomplete GIM (type II and type III) based on the profile of mucins that are secreted^19^. Both AG and GIM are caused by *Helicobacter pylori* infection and, less commonly, autoimmune gastritis.

The severity and topographic distribution of AG and GIM are associated with GC risk. GIM and AG are staged histologically using the Operative Link on Gastric Intestinal Metaplasia (OLGIM) and Operative Link on Gastritis Assessment (OLGA), respectively, and progress in severity from Stage 0 to IV^20^. The staging is derived from mapping biopsies of the antrum, incisura and corpus using the updated Sydney protocol.

GIM is an independent risk factor for GC with a pooled incidence rate of progression to GC of 12.4 GC cases per 10,000 patients-years’ time. The cumulative incidence of GC at 3 years, 5 years and 10 years is 0.4%, 1.1% and 1.6%, respectively^21^. The risk of GC is 2 to 5-fold higher in subgroups with a first-degree relative with GC, incomplete GIM, specifically type III, corpus GIM and OLGIM Stage III and IV^22–25^.

A recent prospective cohort with standardised mucosal sampling demonstrated that OLGIM II also carries an increased risk of a combined outcome of high-grade dysplasia and adenocarcinoma (EGN). Age-adjusted rates of EGN for OLGIM I, II and III/IV were 22, 109 and 544 per 100,000 person-years. The progression to EGN took longer with OLGIM II compared to OLGIM III/IV (median 51 vs 23 months). Endoscopic surveillance with intervals stratified by risk resulted in a timely diagnosis of GC with no late-stage GC^26^.

The risk of malignant progression in AG is estimated to range from 0.1-0.3% per year, but it may be higher with more severe and extensive AG (higher OLGA/OLGIM stages) and concomitant GIM.

The presence of dysplasia and EGC is the threshold for endoscopic intervention. High-grade dysplasia (HGD) has a 60–85% rate of malignant progression or synchronous cancer. As biopsy samples are small and thus often unable to differentiate HGD from GC, most visible dysplastic lesions are upstaged to GC following endoscopic resection^27^. The presence of gastric dysplasia is also associated with synchronous GC in up to 30% of cases. While the natural history of low-grade dysplasia (LGD) is less clear, 25% of LGD is upstaged to either HGD GC following resection^27–29^. Hence, dysplastic lesions require resection for definitive staging, as GC may already be present.

The management of dysplasia and early gastric cancer has been advanced by the adoption of endoscopic submucosal dissection (ESD), which provides definitive histological staging, curative treatment for early gastric cancer in the absence of risk factors for lymph node metastases and comparable outcomes to surgery in well-selected patients^30,31^.

In addition, the detection of dysplastic lesions, which are often subtle, has been greatly aided by the development of advanced endoscopic imaging techniques. These techniques - dye-based chromoendoscopy and electronic chromoendoscopy - improve detection by enhancing mucosal contrast and delineating mucosal surface irregularities. Dyes differentially stain normal and neoplastic mucosa and delineate subtle mucosal irregularities. Electronic chromoendoscopy, such as narrow-band imaging (NBI), relies on optical filters to differentiate normal and neoplastic mucosa based on their different optical signatures^32–34^.

## Surveillance practice recommendation

Endoscopic surveillance is thus performed with two main objectives - to detect neoplastic lesions that may be candidates for endoscopic intervention and to stage the severity of GIM/AG to guide surveillance intervals.

Universal surveillance is not recommended as GIM is prevalent - occurring in 14–37% of routine endoscopic biopsies - and the absolute risk of GC is low^35–37^. High-risk subgroups are the targets of endoscopic surveillance. Specific recommendations for surveillance intervals vary among expert guidelines. These largely target patients with GIM who have additional risk factors such as incomplete GIM, extensive GIM, OLGIM/II/IV, or family history of GC as well as patients with overall increased GC risk, such as ethnic minorities in Western populations and immigrants from high-incidence regions. While the details differ, surveillance endoscopy is generally recommended every 3–5 years^38–41^. More recent data supports 2-yearly endoscopies for certain high-risk individuals^26^. Routine surveillance is generally not recommended in most patients with GIM limited to the distal stomach.

## Limitations of biopsy-based surveillance

While surveillance guidelines are based on biopsy-based risk stratification, the use of histological classifications to stratify neoplastic risk has yet to find widespread routine adoption. While OLGIM is a more reproducible determinant of risk, with high interobserver agreement compared to OLGA, both OLGIM/OLGA staging have limited utility when only a few biopsies are available for examination^42,43^. The severity may also be downstaged in the absence of an incisura biopsy. The limited availability of biopsies may be overcome by subtyping GIM instead, as incomplete GIM increases GC risk. Furthermore, it is now recognised that incomplete GIM is prevalent not just in individuals with extensive GIM, but even in individuals with OLGIM Stage I. Hence, OLGIM staging without GIM subtyping may lead to high-risk patients being misclassified and thus being missed for surveillance^44^. However, GIM subtyping is not routinely performed due to concerns related to reproducibility from operator-dependent techniques.

A scale for endoscopic grading of intestinal metaplasia (EGGIM) using NBI has been developed and validated to simplify risk stratification without recourse to routine mapping biopsies^45^. EGGIM identifies OLGIM III/IV, and EGGIM grading is associated with the risk of GC^46^. EGGIM shows promise in providing real-time risk stratification without recourse to routine biopsies. This may also allow for a more global assessment of GIM, which may be patchy and multifocal and thus missed on random biopsies, and raises the intriguing possibility of trending dynamic changes in the extent of GIM on serial endoscopies. However, it requires prospective validation as a predictor of GC risk. It would also require additional procedural time, and adherence is debatable outside academic settings.

On a more fundamental note, GIM surveillance is premised on the identification of a small subset of high-risk patients within the GIM population using a systematic mapping or biopsy protocol and meticulous examination to detect subtle neoplastic change. Low adherence to the biopsy protocol or a non-restrictive approach that expands surveillance to all GIM would arguably negate the effectiveness of surveillance.

It is also instructive to note that GIM is the precursor to intestinal-type GC but not diffuse-type GC. Hence, the latter may not be amenable to a preventive strategy focused on GIM.

## Dilemma of incidental GIM

As AG and GIM are asymptomatic and manifest subtly on endoscopy, they are often diagnosed incidentally on non-targeted endoscopic biopsies for an unrelated indication where mapping biopsies may not have been performed *a priori*. Hence, in patients with an unexpected diagnosis of GIM on routine biopsies, questions arise as to the adequacy of the original exam for surveillance as well as for the detection of neoplastic lesions.

In these instances, it is unclear if a routine second-look endoscopic examination at a short interval is justified for risk stratification and detection of missed neoplasia. Such a consideration is tempered by the observation that the risk of GC at one year following a diagnosis of GIM is estimated to be low (5 per 1000 persons)^21^. Yet the prevalence of GIM on routine endoscopic biopsies makes this a commonly encountered clinical dilemma that requires further elucidation. The answer may lie - at least in part - in a paradigm shift in the philosophical approach towards diagnostic gastroscopy that is already underway.

## Should every endoscopic examination be regarded as an opportunity to screen for GC?

Increasingly it is advocated that every routine gastroscopy be regarded as an opportunity to screen for GC^47^. Even in the setting of population-based mass endoscopic screening, the majority of early GCs are actually detected on endoscopies performed outside screening programs. This underscores the importance of a careful diagnostic examination that actively seeks out subtle neoplastic lesions as the norm^12^. In this paradigm, a purposeful, systematic, high-quality examination with the intent to detect early neoplasia becomes the norm for all routine diagnostic endoscopies. This would find common cause with colorectal cancer, where the adequacy of a diagnostic colonoscopy is largely determined by quality metrics related to adenoma detection.

Such an approach would entail a significant change in mindset and practice. Would this philosophy find traction where GC is an uncommon occurrence? Furthermore, EGN is subtle and easily missed on endoscopy. Training in systematic examination and the detection and characterization of subtle endoscopic features using adjunctive endoscopic imaging would have to be incorporated into the endoscopy training curriculum^48^. In addition, while definitions for a high-quality examination have been proposed, robust evidence-based quality indicators for upper endoscopy in GC prevention remain a largely unmet need.

## Gastric Intestinal Metaplasia, the new Barrett’s esophagus?

Perhaps the most significant change is that growing calls for endoscopic surveillance render GIM, a largely asymptomatic entity and a suboptimal determinant of GC risk, a de facto disease. It effectively reframes the focus of interest from GC to GIM. In this regard, it is instructive to reflect on some of the pitfalls encountered with another metaplastic epithelium within the digestive tract that is subject to surveillance – Barrett’s esophagus (BE).

A diagnosis of BE increases insurance premiums and limits access to insurance coverage^49^. Patients overestimate their short-term risk of cancer. The diagnosis of a precancerous condition often prompts an activist approach - “an urge to do something” - which is counterproductive in the vast majority of patients who may never develop cancer and die of unrelated causes^50^. The ensuing psychological stress of surveillance and the reduced quality of life is not insignificant.

How do we navigate a similar path with gastric GIM and reach a different outcome? Even as the global incidence of GC continues to decline? Is GIM simply a convenient surrogate on which to base practice recommendations when what truly matters is the prevention of GC? While current GIM surveillance recommendations target high-risk subgroups, would a less restrictive “real-world” surveillance practice result in more “GIM sufferers”?

While GIM is a common endoscopic finding whose management needs clarification, it is not the focus of prevention. Cancer is.

## Let’s not forget *H. pylori* (HP)

Unlike Barrett’s adenocarcinoma and colorectal cancer, GC has a preventable underlying cause - *H. pylori* infection^51^.

HP eradication is a recommended primary preventive strategy for GC^52^. It prevents cancer incidence and mortality^53^. HP eradication reduces the risk of GC in asymptomatic infected individuals and in individuals with a first degree relative with GC^54^. It reduces the risk of metachronous GC in subjects undergoing curative endoscopic resection^53^. The benefits of HP treatment on GC incidence and mortality persist after 20 years^55^. Similarly, HP treatment has a long-term beneficial effect on the progression of precancerous lesions^55,56^. Mass eradication in a high-risk population has been demonstrated to decrease GC incidence^57^.

While mass HP eradication in low-intermediate risk settings is unproven, the logistics are considerable, rising antibiotic resistance complicates eradication strategies, HP prevalence is uneven, the incidence is falling, and the benefits of eradication in subjects with pre-existing precancerous change are attenuated, HP eradication addresses the root cause of GC and remains a proven strategy for GC prevention^58,59^. While HP eradication and GIM surveillance are not mutually exclusive, proven strategies should be prioritised.

## Conclusion

Endoscopic screening and surveillance have expanded the armamentarium available to combat the scourge of GC. Yet they also pose new questions that will require definitive answers to support the case for endoscopy-based GC preventive strategy.
